# Intermittent Hypoxia Rewires the Liver Transcriptome and Fires up Fatty Acids Usage for Mitochondrial Respiration

**DOI:** 10.3389/fmed.2022.829979

**Published:** 2022-02-18

**Authors:** Jonathan Gaucher, Guillaume Vial, Emilie Montellier, Maëlle Guellerin, Sophie Bouyon, Emeline Lemarie, Véronique Pelloux, Anne Bertrand, Karin Pernet-Gallay, Frederic Lamarche, Anne-Laure Borel, Claire Arnaud, Elise Belaidi, Karine Clément, Diane Godin Ribuot, Judith Aron-Wisnewsky, Jean-Louis Pépin

**Affiliations:** ^1^Hypoxia and PhysioPathology (HP2) Laboratory, INSERM U1300, CHU Grenoble-Alpes, University Grenoble-Alpes, Grenoble, France; ^2^CNRS 5309, INSERM U1209, Institute for Advanced Biosciences, University Grenoble-Alpes, Grenoble, France; ^3^Nutrition and Obesities, Systemic Approaches, NutriOmics, Laboratory, Sorbonne University, Paris, France; ^4^Nutrition Department, CRNH Ile de France, Assistance Publique Hôpitaux de Paris, Pitie-Salpêtrière Hospital, Paris, France; ^5^INSERM U1216, Grenoble Institute of Neurosciences, University Grenoble-Alpes, Grenoble, France; ^6^Laboratory of Fundamental and Applied Bioenergetics (LBFA), INSERM U1055, University Grenoble Alpes, Grenoble, France

**Keywords:** sleep apnea, intermittent hypoxia (IH), liver, transcriptome, mitochondria, Nuclear Respiratory Factor (NRF)

## Abstract

Sleep Apnea Syndrome (SAS) is one of the most common chronic diseases, affecting nearly one billion people worldwide. The repetitive occurrence of abnormal respiratory events generates cyclical desaturation-reoxygenation sequences known as intermittent hypoxia (IH). Among SAS metabolic sequelae, it has been established by experimental and clinical studies that SAS is an independent risk factor for the development and progression of non-alcoholic fatty liver disease (NAFLD). The principal goal of this study was to decrypt the molecular mechanisms at the onset of IH-mediated liver injury. To address this question, we used a unique mouse model of SAS exposed to IH, employed unbiased high-throughput transcriptomics and computed network analysis. This led us to examine hepatic mitochondrial ultrastructure and function using electron microscopy, high-resolution respirometry and flux analysis in isolated mitochondria. Transcriptomics and network analysis revealed that IH reprograms Nuclear Respiratory Factor- (NRF-) dependent gene expression and showed that mitochondria play a central role. We thus demonstrated that IH boosts the oxidative capacity from fatty acids of liver mitochondria. Lastly, the unbalance between oxidative stress and antioxidant defense is tied to an increase in hepatic ROS production and DNA damage during IH. We provide a comprehensive analysis of liver metabolism during IH and reveal the key role of the mitochondria at the origin of development of liver disease. These findings contribute to the understanding of the mechanisms underlying NAFLD development and progression during SAS and provide a rationale for novel therapeutic targets and biomarker discovery.

## Graphical Abstract

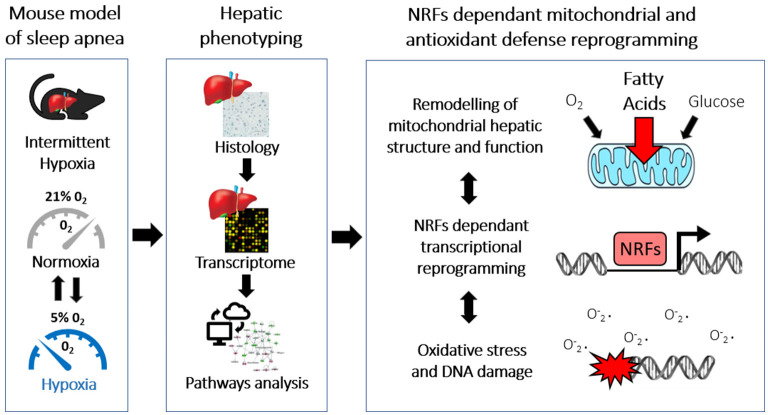

## Highlights

- IH increases expression of electron transport chain (ETC) genes in the liver.
- IH responsive genes in the liver show enrichment for Nuclear Respiratory Factors (NRFs) regulatory elements.
- IH alters hepatic mitochondrial structure and function.
- IH enhances hepatic fatty acid utilization for mitochondrial respiration.
- IH is accompanied with oxidative stress and DNA damage in the liver.

## Introduction

Sleep Apnea Syndrome (SAS) is one of the most common chronic diseases affecting nearly one billion people worldwide (1). SAS is characterized by repetitive complete (apnea) or partial (hypopnea) collapse of the upper airway during sleep. The repetitive occurrence of these abnormal respiratory events generates cyclical desaturation-reoxygenation sequences known as intermittent hypoxia (IH) (2). Among the metabolic consequences of SAS, it has been established by experiments in animals and human clinical studies that SAS is an independent risk factor for the development and progression of non-alcoholic fatty liver disease (NAFLD), accelerating the transition from steatosis to non-alcoholic steatohepatitis (NASH) and progression to fibrosis (3–7).

NAFLD is the most common cause of chronic liver disease in Western countries and is also predicted to become the most frequent indication for liver transplantation by 2030 (8, 9). There is growing evidence that NAFLD is a multisystem disease, affecting extra-hepatic organs, generating cardiovascular complications and early mortality (8, 10, 11). There is currently no approved pharmacotherapy for NAFLD, although several classes of compounds are in advanced stages of development. Therefore, a better understanding of the specific molecular mechanisms linking IH and liver disease progression will contribute to the development of new drugs and management pathways in the field.

The principal goal of this study was to decipher the molecular mechanisms of liver injury induced by IH. To address this question, using a unique model of mice exposed to IH (12), first, we employed unbiased high-throughput transcriptomics to generate hypotheses and provide a comprehensive analysis of liver metabolism under IH. This revealed that IH reprograms hepatic transcription and shifts metabolic mitochondrial function, promoting fatty acids as substrate. In parallel, the deregulation of the balance between oxidative stress and antioxidant defense is linked to the increase in hepatic ROS production and DNA damage during IH.

## Materials and Methods

### Animal Handling

All experimental procedures were carried out in accordance with European Directive 2010/63/UE. They were reviewed by the Institutional Ethics Committee for Animal Care and Use (Cometh 12) and authorized by the French Ministry of Research (APAFIS# 15156-2018051615245109).

**Figure 1:**
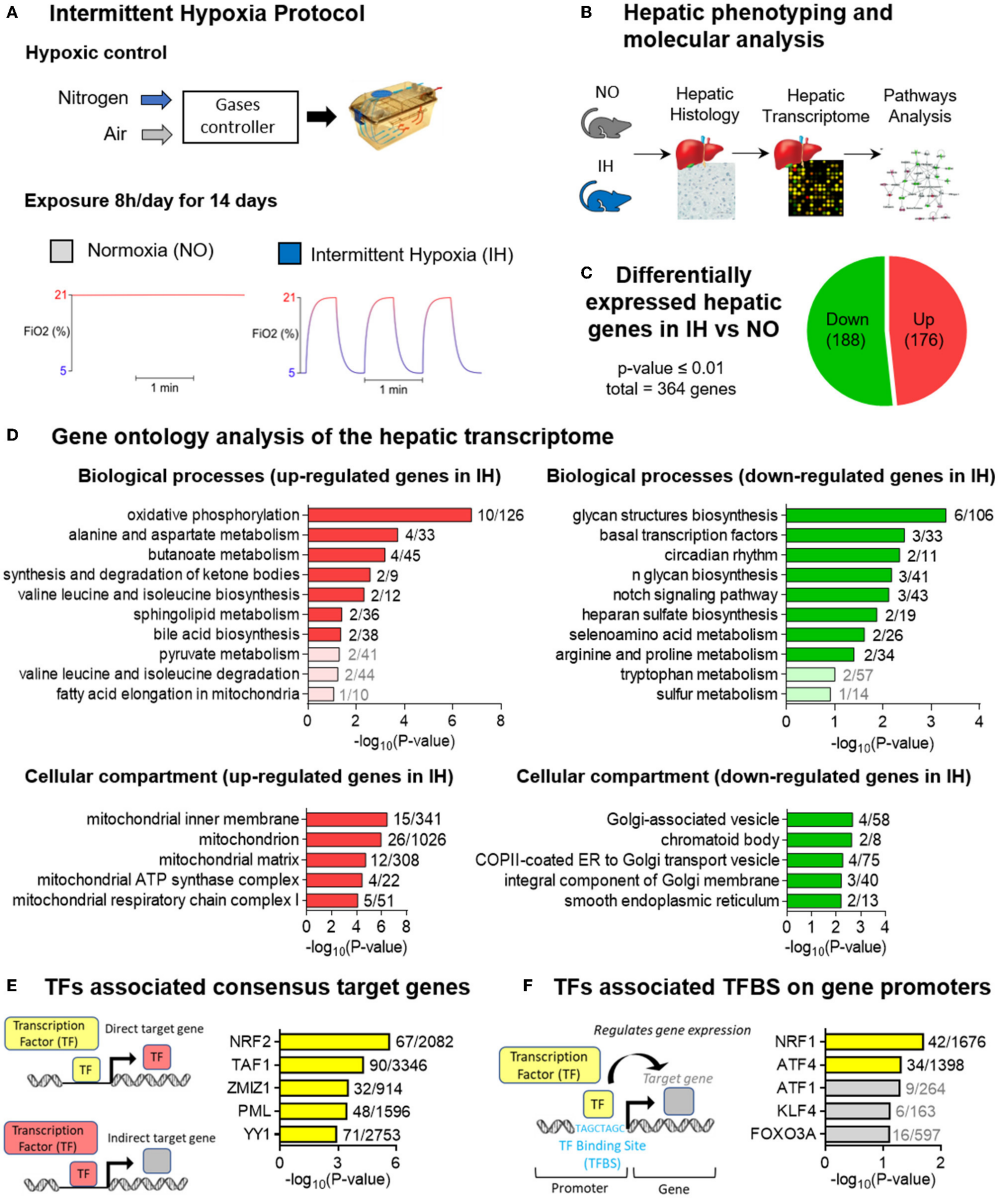
The hepatic transcriptomic signature generated by intermittent hypoxia. **(A)** Schema outlining the experimental design of intermittent hypoxia exposure. Male mice were directly exposed in their cages to 2 weeks of Intermittent Hypoxia (IH: 1-min cycles alternating 30s of 5% FiO_2_ and 30s of 21% FiO_2_, 8h/day during sleep) or Normoxia (NO: identical 1-min cycles alternating 21% FiO_2_, 8h/day during sleep, providing noise and air turbulence due to gas circulation similar to those of IH exposure). **(B)** Schema illustrating the experimental design for hepatic phenotyping and molecular analysis. **(C)** Pie chart representing the number of differentially expressed genes due to IH. Upregulated genes are shown in red and downregulated genes are shown in green. Genes were selected using a *P* ≤ 0.01 indicating a significant difference between NO and IH (*n* = 7–8 biological replicates per group). **(D)** Gene ontology (GO) analysis showing the top 10 biological processes enriched in upregulated genes upon IH (top left) and downregulated genes upon IH (top right) and the top five cellular compartments enriched in upregulated genes upon IH (bottom left) and downregulated genes upon IH (bottom right). The number of dysregulated genes in our transcriptome over the total number of genes for each GO category is indicated on the graph. **(E)** Transcription factor (TFs) analysis showing the top five enriched TFs associated with target genes that are present in the ENCODE database. For TFs with data from multiple experiments, set intersection was applied to obtain consensus. The number of dysregulated genes in our transcriptome over the total number of consensus target genes associated with each TF is indicated on the graph. **(F)** TFs analysis showing the top five enriched TFs associated with TF binding sites (TFBS) detected at the promoter of genes that are present in TRANSFAC and JASPAR databases. The number of dysregulated genes in our transcriptome over the total number of TFBS associated with each TF target gene is indicated on the graph.

Sixteen-week-old male mice (C57BL/6JRj; Janvier Labs, France) were housed 5 per cage with *ad libitum* access to food (LASQC diet, Rod 16, Altromin international) and water. The animal facility was on a 12 h light/12 h dark cycle (light from 8 am to 8 pm) at 22°C ± 2°C. Mice were randomly assigned to either intermittent hypoxia (IH) or normoxia (NO) and directly exposed in their housing cages (Tecniplast) to 14 days of NO or IH, 8h per day during their sleeping period (IH from 8 am to 4 pm and NO the rest of the day) (Figure 1A).

The IH stimulus consisted of 60 sec cycles alternating 30 sec of hypoxia (hypoxic plateau at 5% FiO_2_) and 30 sec of NO (normoxic plateau at 21% FiO_2_). Normoxic mice were exposed to similar air-air cycles in order to avoid bias from noise and turbulence related to gas flow. On day 15 the mice were sacrificed by decapitation, the livers dissected and immediately frozen in liquid nitrogen until analysis.

### DNA and RNA Extraction

For genomic DNA (gDNA) extraction, 50 mg of frozen liver tissues were thawed in Digestion Buffer (Tris 50mM pH8, EDTA 5mM, NaCl 200 mM, SDS1%) and digested overnight at 56°C after adding 20 mg/ml proteinase K (#09-0912, Euromedex). After centrifugation at 20,000 g for 15 min at 20°C, the supernatant was collected and same volume of phenol-chloroform solution was added (#A0889,0100 AppliChem, BioChemica). After centrifugation at 20,000 g for 2 min at 20°C, the upper aqueous phase was collected and 10 mg/ml RNase A was added (#EN0531, Thermoscientific). After 15 min incubation at 20°C, 3 volumes of ethanol 100% were added (#4146082, Carlo Erba Reagents) for 1 volume of upper aqueous phase. After centrifugation at 20,000 g for 15 min at 20°C the DNA pellet was washed twice in ethanol 70% (#528170, Carlo Erba Reagents), air dryed and resuspended in ultrapure DEPC treated water (#75-002-4, Thermo Fisher Scientific). DNA sample was stored at −80°C after flash-freezing in liquid nitrogen.

For RNA extraction, 50 mg of frozen liver tissues were homogenized in TRIzol Reagent (Invitrogen, 15596026) and RNA was extracted after precipitation with isopropanol and ethanol washes according to the manufacturer protocol. RNA sample was stored at −80°C after flash-freezing in liquid nitrogen.

### Reverse Transcription and Quantitative Real Time PCR Analysis

One μg of RNA was reverse transcribed to cDNA using iScript complementary DNA (cDNA) synthesis kit (Bio-Rad Laboratories, 1708840), according to the manufacturer's protocol.

cDNA or DNA were used for quantitative real-time PCR (RT-qPCR) using SsoAdvanced SYBR Green Supermix kit (Bio-Rad Laboratories, 1725270) according to the manufacturer's protocol. Gene expression was normalized to beta actin.

Primer sequences used for analysis are listed in Supplementary Primers List.

### Microarray Experiment

RNA concentration and integrity were assessed with the Agilent 2100 Bioanalyzer (Agilent Technologies) and amplified with the Illumina RNA amplification kit according to the manufacturer's protocol (Ambion) to obtain biotin-labeled complementary RNA from 250 ng total RNA. Hybridization processes were performed with Illumina MouseRef-8 v2.0 Expression BeadChip (Illumina Inc). Hybridized probes were detected with cyanin-3-streptavidin (1 mg/mL; Amersham Biosciences, GE Health Care) and scanned using an Illumina BeadArray Reader. Raw data were extracted with GenomeStudio 2011.1 software using the default settings and without any additional normalization.

The difference in gene expression between the two conditions (NO or IH) was determined using SAM software (Significant Analysis of Microarray, Stanford University, CA, USA, https://statweb.stanford.edu/~tibs/SAM/), which provides a list of significant genes and an estimate of the false discovery rate (FDR) representing the percentage of genes that could be identified by chance. The FDR was tested at 5%.

### Data Availability

The gene expression data reported in this paper are available in the National Center for Biotechnology Information Gene Expression Omnibus (https://www.ncbi.nlm.nih.gov/geo/). The accession number is GSE104128.

### Statistical Analysis

Statistical analyses are detailed for each experiment in the figure legend. Heat maps were generated by R package “pheatmap”. Graphs were depicted using Microsoft Excel, GraphPad Prism and Affinity Designer.

### Functional Analysis

Functional analysis of the hepatic transcriptome was performed as previously described (13, 14). Briefly, the Database for Annotation, Visualization and Integrated Discovery (DAVID) and EnrichR pathway analysis tools were used to identify Gene Ontology (GO) terms (biological process and cellular compartments) and transcription factor binding site enrichment.

### Electron Microscopy

Liver pieces were fixed with glutaraldehyde 2% and paraformaldehyde 2% in phosphate buffer 0.1 M pH7.4 during 24 h at 20°C. Tissue was then washed with phosphate buffer 0.1 M pH7.4 and stained with Osmium Tetroxide 1% and Cacodylate 0.1 M pH7.2 during 1 h at 4°C. After extensive washes with water, tissues were further stained with Uranyl Acetate 1% pH4 during 1 h at 4°C before being dehydrated through graded ethanol solutions (30–60–90–100–100–100%). Tissues were then infiltrated with a mix of 1/1 epon/ethanol 100% during 1 h and went through several baths of fresh epon (Flukka) during 3 h. Finally, samples were embedded in fresh epoxy resin and left to polymerize during 72 h at 60°C. Ultrathin sections of tissue were cut with an ultramicrotome (Leica), post-stained with Uranyl Acetate 5% and Lead Citrate 0.4%. Tissue sections were observed with a transmission electron microscope (acceleration voltage: 80 k) (JEOL 1200EX) andimages were acquired with a digital camera (Veleta, SIS, Olympus). Themorphometric analysis was performed with iTEM software 7 (Olympus).

### Determination of Protein Concentration

Protein concentration was determined using the bicinchoninic acid (BCA) assay using bovine serum albumin (BSA) as a standard according to the manufacturer's protocol (Pierce).

### Determination of Mitochondrial Enzymatic Activities

Mitochondrial complexes activities were assessed on three times frozen and thawed purified mitochondria in order to alter the membranes and allow the access of the substrates to the enzymes.

Complex I: Rotenone-sensitive NADH-ubiquinone oxidoreductase (EC 1.6.5.3) activity was quantified using decylubiquinone 100 μM as electron acceptor and NADH 200 μM as a donor, in KH_2_PO_4_/K_2_HPO_4_ buffer at 10 mM pH7.5 containing BSA 3.75 mg/mL, KCN 2 mM, antimycin-A 7.5 μM. NADH oxidation was measured at 340 nm, before and after the addition of rotenone 4 μM to allow the calculation of the rotenone-sensitive specific activity which is characteristic of complex I.

Complex II: Succinate-ubiquinone reductase (EC 1.3.5.1) activity was quantified by measuring the decrease in absorbance at 600 nm due to the reduction of DCIP 100 μM. The measurement was performed in KH_2_PO_4_/K_2_HPO_4_ buffer 50 mM pH 7.5 in the presence of succinate 30 mM, decylubiquinone 100 μM, rotenone 2 μM and KCN 2 mM.

Complex III: Coenzyme Q—Cytochrome C—oxidoreductase activity (EC 1.10.2.2) was quantified by measuring the increase in absorbance at 550 nm due to the reduction of Cytochrome C at 100 μM. The measurement was performed in KH_2_PO_4_/K_2_HPO_4_ at 50mM pH7.5 in the presence of decylubiquinone 100 μM previously reduced by dithionite, EDTA 50 μM, KCN 1 mM. The specific activity was calculated by subtracting the activity obtained before and after addition of antimycin A at 5 μg/ml.

Complex IV: Cytochrome C oxidase (EC 1.9.3.1) activity was quantified by measuring oxidation of Cytochrome C 100 μM at 550 nm in KH_2_PO_4_/K_2_HPO_4_ buffer at 50 mM pH7.0.

Citrate synthase activity (EC 2.3.3.1) was determined in liver tissue as previously described (15).

### Analysis of Mitochondrial Oxygen Consumption

Hepatic mitochondria isolation and analysis was performed as previously described (16). Briefly, samples went through differential centrifugation procedure in Tris-HCl 20 mM pH7.4, sucrose 250 mM, EGTA 1 mM. The rate of mitochondrial oxygen consumption (JO_2_) was measured at 30°C using a Clark-type O_2_ electrode in a 1 mL-chamber filled with respiration buffer: Tris-HCl 20 mM pH 7.2, KCl125 mM, Pi-Tris10 mM, EGTA 0.1 mM, and using 1 mg/mL of mitochondrial proteins. Measurements were made in the presence of either glutamate 5 mM and malate 2.5 mM or succinate 5 mM or palmitoyl-carnitine 55 μM as substrates (state 2), after the addition of ADP 1 mM (state 3), followed by the addition of oligomycin 0.25 mg/mL (state 4).

### TUNEL Assay

The analysis of DNA fragmentation was evaluated by applying terminal deoxynucleotidyl transferase-mediated dUTP nick end-labeling (TUNEL) assay according to the manufacturer's protocol (Abcam). Briefly 5 μm thick paraffin embedded liver tissues were processed for TUNEL assay and the Axioscan fluorescent microscope (Zeiss) was used to visualize TUNEL-positive cells and nuclei counter-staining with 4,6-diamidino-2-phenylindole (DAPI). The TUNEL assay was performed on three biological replicates per group and four areas per biological replicate were analyzed using Image J® software (National Institutes of Health). TUNEL-positive cells were expressed as the percentage of liver area.

### Dihydroethidium (DHE) Staining

Cells are permeable to Dihydroethidium (DHE), which, upon reaction with a superoxide anion (O_2_-), forms ethidium bromide (a red fluorescent product) that intercalates in DNA. Frozen livers were cryosectioned at 10 μm thick, collected onto Superfrost plus slides (Dutscher France) and allowed to air dry for 15 min at room temperature. Slides were stained with 10 μM DHE (Sigma-Aldrich) for 30 min at 37°C (PBS was used as control) in a dark moist chamber. After washing, cover slips were added and the fluorescent signal was recorded using confocal microscopy (Zeiss, LSM510 Meta confocal microscope) and analyzed with ImageJ software. DHE-positive cells were expressed as the percentage of liver area.

### Immunohistochemistry

Protein detection by immunohistochemistry was performed as previously described (17). Briefly, glass slides (Superfrost Plus, Thermo Fischer) with 10 μm slices of paraffin embedded liver were rehydrated and processed for antigen retrieval with citrate buffer pH6 (C999, Sigma Aldich). Then, samples went into 3% hydrogen peroxide (H1009, Sigma Aldrich), to quench endogenous peroxidase activity and successively incubated with avidin-biotin blocking kit (SP-2001, Vector Laboratories), 5% goat serum (Vector Laboratories) and NRF2 rabbit primary antibody (ab31163, Abcam). After PBS washes, slides were incubated with biotinylated goat anti rabbit secondary antibody (Vector Laboratories) and processed according to the manufacturer's protocol (PK-6101, Vectastain Elite ABC kit peroxidase, Vector Laboratories). Then colorimetric substrate was added (E109, HistoGreen, Novus Biological) and slices were counterstained with Mayer hematoxylin (51275, Sigma Aldrich). Vectamount permanent medium (Vectorlab) was used to fix the coverslips on the slides. Images were then acquired using an Axio Scan microscope (Zeiss) and Zen® software (Zeiss).

## Results

### Unique Hepatic Transcriptomic Signature Related to IH

We investigated whether 14 days of IH alters the hepatic transcriptome (Figure 1B). Bioinformatic analysis revealed that IH significantly alters the expression of 364 genes. Among them, 176 were upregulated and 188 downregulated by IH (Figure 1C). Gene ontology analysis highlighted changes in distinct biological processes and cellular compartment. Both oxidative phosphorylation and mitochondria functional modules were overrepresented in the upregulated genes, whereas glycan biosynthesis and Golgi functions were prominent among those that were downregulated (Figure 1D).

### NRF-Dependent Hepatic Transcription in Response to IH

To decipher the transcriptional pathways rewired by IH, transcription factor binding sites (TFBS) and consensus target genes analysis were performed on significantly altered hepatic transcripts using EnrichR to identify associated transcription factors (TFs) (Figures 1E,F). Nuclear Respiratory Factor 2 (NRF2) consensus target genes (Figure 1E) and Nuclear Respiratory Factor 1 (NRF1) binding sites were highly enriched in our dataset (Figure 1F). NRF1 and NRF2 are cap-n-collar basic leucine zipper (CNC-bZIP) transcription factors that regulate mitochondrial respiration and antioxidant defenses (18–20), there was thus a strong rationale to target mitochondria for subsequent experiments.

### IH Impacts the Expression of Hepatic Mitochondrial Electron Transport Chain Subunits Without Altering Individual Complex Activities

**Figure 2:**
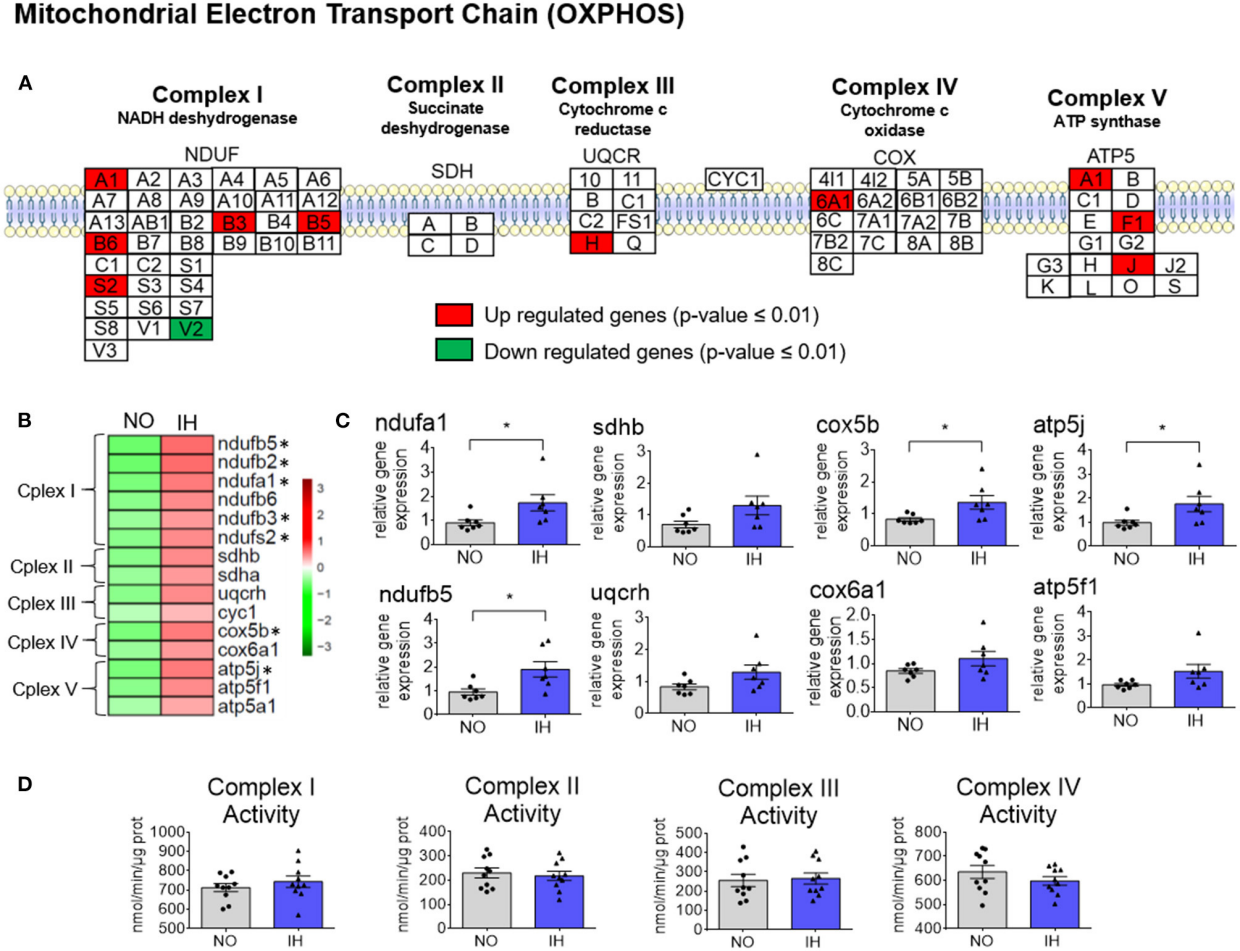
Rewiring of the mitochondrial electron transport chain (ETC) complexes (Cplex) due to IH. **(A)** Scheme representing the subunits of the five complexes of the mitochondrial ETC. ETC components with significantly dysregulated expression according to the transcriptome are highlighted (*P* ≤ 0.01). Upregulated genes are highlighted in red and downregulated genes are highlighted in green. **(B)** Heatmap of representative gene expression profiles associated with ETC determined by quantitative real-time PCR. Gene expression was normalized to beta-actin and presented as mean + standard error of mean (SEM, *n* = 7 biological replicates per group). Significance was calculated using a Student's *t* test and *indicates *p*-value cut-offs of 0.05. **(C)** Selected expression profiles of genes belonging to the ETC determined by quantitative real-time PCR. Gene expression was normalized to beta-actin and presented as mean + standard error of mean (SEM, *n* = 7 biological replicates per group). Significance was calculated using Student's *t* test and *indicates *p*-value cut-offs of 0.05. **(D)** Enzymatic activity of ETC complexes in isolated mitochondria from NO and IH mouse livers. Activity was normalized to the amount of proteins from isolated mitochondria and presented as mean + standard error of mean (SEM, *n* = 10 biological replicates per group).

To better understand the impact of IH on mitochondrial physiology, we focused our attention on the mitochondrial genes that were differentially expressed in the hepatic transcriptome. Most of these genes belong to the electron transport chain (ETC) and are upregulated upon IH (Figures 2A,B and Supplementary Figure 2). This is exemplified by the expression profiles of prototypical NRFs target genes such as *Cox5b* (ETC complex IV), *Atp5j* (ETC complex V) and *Tfam* [Figure 2C and Supplementary Figure 2, (21)]. Nonetheless the activity of complex I, II, III and IV was not different between groups (Figure 2D).

### IH Alters Hepatic Mitochondria Morphology and Dynamics

**Figure 3:**
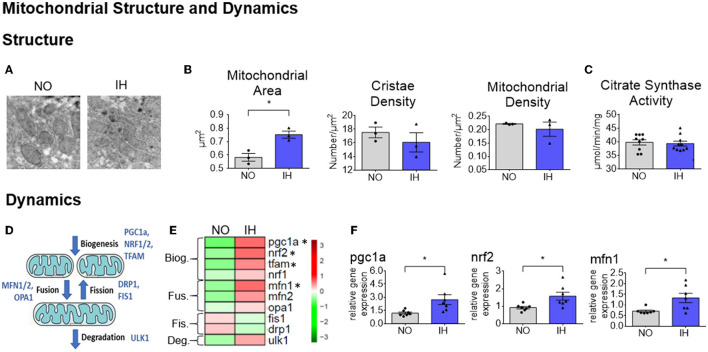
Remodeling of mitochondrial ultrastructure and dynamics due to IH. **(A)** A comparison of representative mitochondria from NO and IH livers by electron microscopy (EM) **(B)** Mitochondrial surface (left panel), cristae density (middle panel) and mitochondrial density in the field (right panel) were quantified (*n* = 3 biological replicates per group) and presented as mean + SEM **(C)** Mitochondrial number was evaluated through the determination of citrate synthase activity (*n* = 9–10 biological replicates per group) and presented as mean + SEM **(D)** Scheme representing the genes involved in mitochondrial dynamics [i.e., biogenesis (Biog.), fusion (Fus.), fission (Fis.) and degradation (Deg.)]. **(E)** Heatmap of representative gene expression profiles involved in mitochondrial dynamics determined by quantitative real-time PCR. Gene expression was normalized to beta-actin and presented as mean + SEM (*n* = 7 biological replicates per group). Significance was calculated using Student's t test and *indicates *p*-value cut-off of 0.05. **(F)** Selected expression profiles of genes involved in mitochondrial dynamics determined by quantitative real-time PCR. Gene expression was normalized to beta-actin and presented as mean + standard error of mean (SEM, *n* = 7 biological replicates per group). Significance was calculated using Student's *t* test and *indicates *p*-value cut-offs of 0.05.

Changes in ETC composition are often associated with morphological adaptations including mitochondrial number, structure or dynamics. We first evaluated the morphological changes of mitochondria using electron microscopy (Figure 3A). We observed no change in mitochondrial density or mitochondrial number (Figures 3B,C and Supplementary Figure 4) but the cross-sectional area of the mitochondria was increased upon IH while the cristae density remained similar between groups (Figure 3B). We then assessed the expression of genes involved in mitochondrial fusion, fission, biogenesis and degradation (Figures 3D–F and Supplementary Figure 3) and found a significant increase in two master regulators of mitochondrial biogenesis and fusion named *Ppargc1a* and *Mfn1* (Figures 3E,F and Supplementary Figure 3). As remodeling of mitochondrial morphology often reflects functional changes, we further assessed mitochondrial function.

### IH Affects Hepatic Mitochondrial Respiration and Substrate Usage

**Figure 4:**
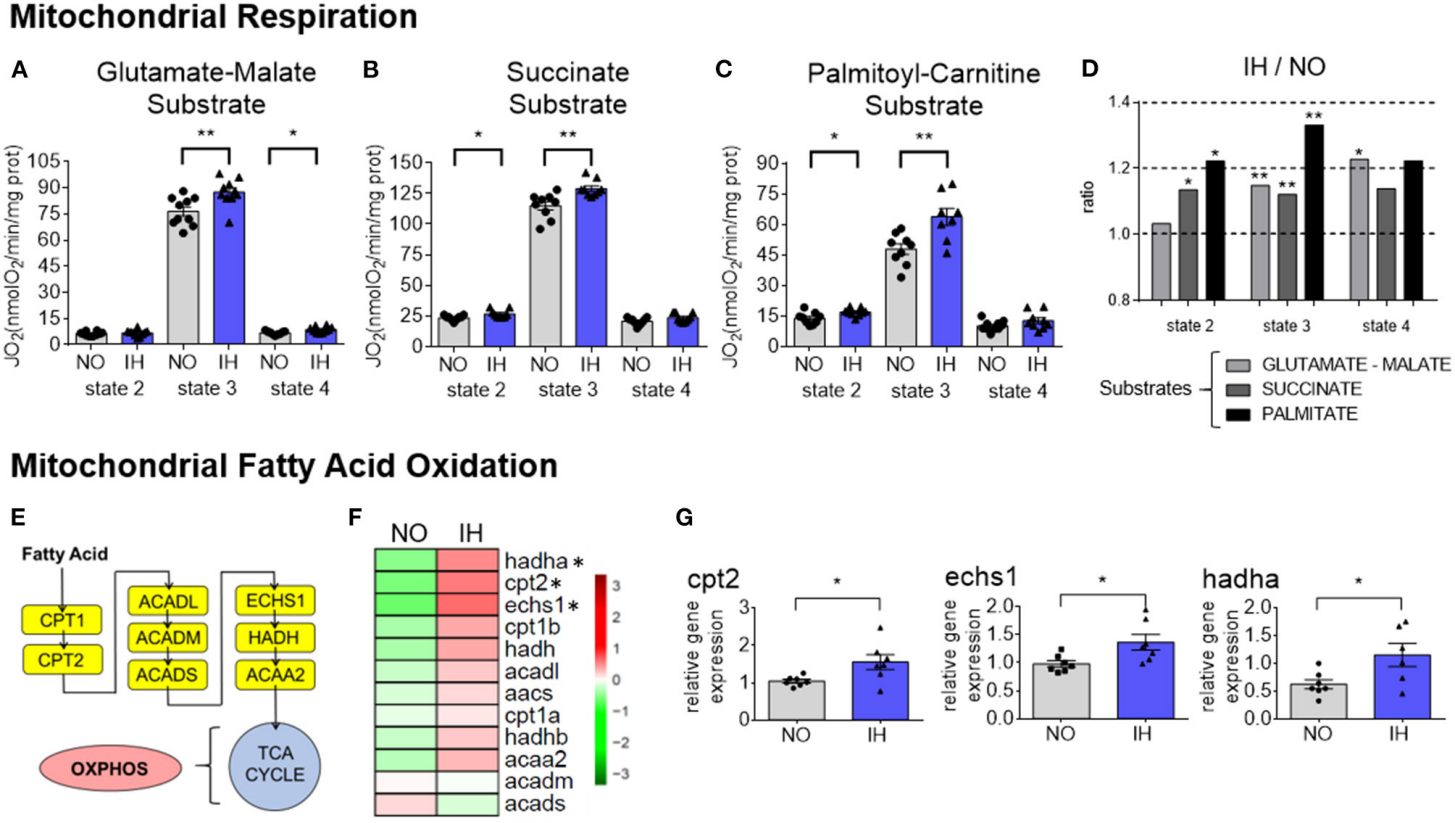
Reprogramming of mitochondrial respiration and fatty acid oxidation. **(A–C)** Mitochondrial oxygen consumption with different substrates in NO and IH livers. Mitochondrial respiration is presented as mean + SEM (*n* = 9–10 biological replicates per group). Significance was calculated using Student's *t* test and * and **indicate *p*-value cut-offs of 0.05 and 0.01, respectively. **(D)** IH over NO ratio of mitochondrial oxygen consumption obtained in **(A–C)**. **(E)** Scheme representing the genes involved in mitochondrial fatty acid oxidation steps. **(F)** Heatmap of representative gene expression profiles involved in mitochondrial fatty acid oxidation determined by quantitative real-time PCR. Gene expression was normalized to beta-actin and presented as mean + SEM (*n* = 7 biological replicates per group). Significance was calculated using Student's *t* test and *indicates *p*-value cut-off of 0.05. **(G)** Selected expression profiles of genes involved in mitochondrial fatty acid oxidation determined by quantitative real-time PCR. Gene expression was normalized to beta-actin and presented as mean + SEM (*n* = 7 biological replicates per group). Significance was calculated using Student's t test and *indicates *p*-value cut-off of 0.05.

We investigated how IH affects the respiration rate of freshly isolated hepatic mitochondria in the presence of specific substrates like glutamate and malate, succinate or palmitoyl-carnitine (Figures 4A–C). Succinate and palmitoyl-carnitine state 2 respiration rates were slightly increased by IH whereas an increase in the state 4 respiration rate was only observed in the presence of glutamate/malate. Mitochondrial state-3 (phosphorylating) oxygen consumption was higher in IH mice than in NO mice independent of the substrate used. However, the increase was greater with palmitoyl-carnitine (+33%) than with glutamate/malate (+14%) or succinate (+12%). To compare the variation between IH and NO we calculated the IH over NO ratio for each substrate and each respiration state (Figure 4D). Since this ratio was markedly increased with the palmitoyl-carnitine substrate, this suggests a remarkable ability of the mitochondria to use fatty acids as fuel in IH conditions.

### IH Remodels the Expression of Hepatic Mitochondrial Fatty Acid Oxidation Genes

Mitochondrial fatty acid oxidation (FAO) depends on several enzymes, transporters, and other facilitating proteins (Figure 4E). We thus analyzed the expression of genes belonging to this pathway (Figures 4F,G and Supplementary Figure 2). Among the genes tested we observed a significant increase in *Cpt2*, the rate-limiting transporter for FAO, *Hadha* and *Echs1* (Figure 4G). Altogether these data strongly support an adaptation of liver metabolism to use fatty acids as fuel upon IH exposure.

### IH Induces Hepatic Oxidative Stress and DNA Damage

**Figure 5:**
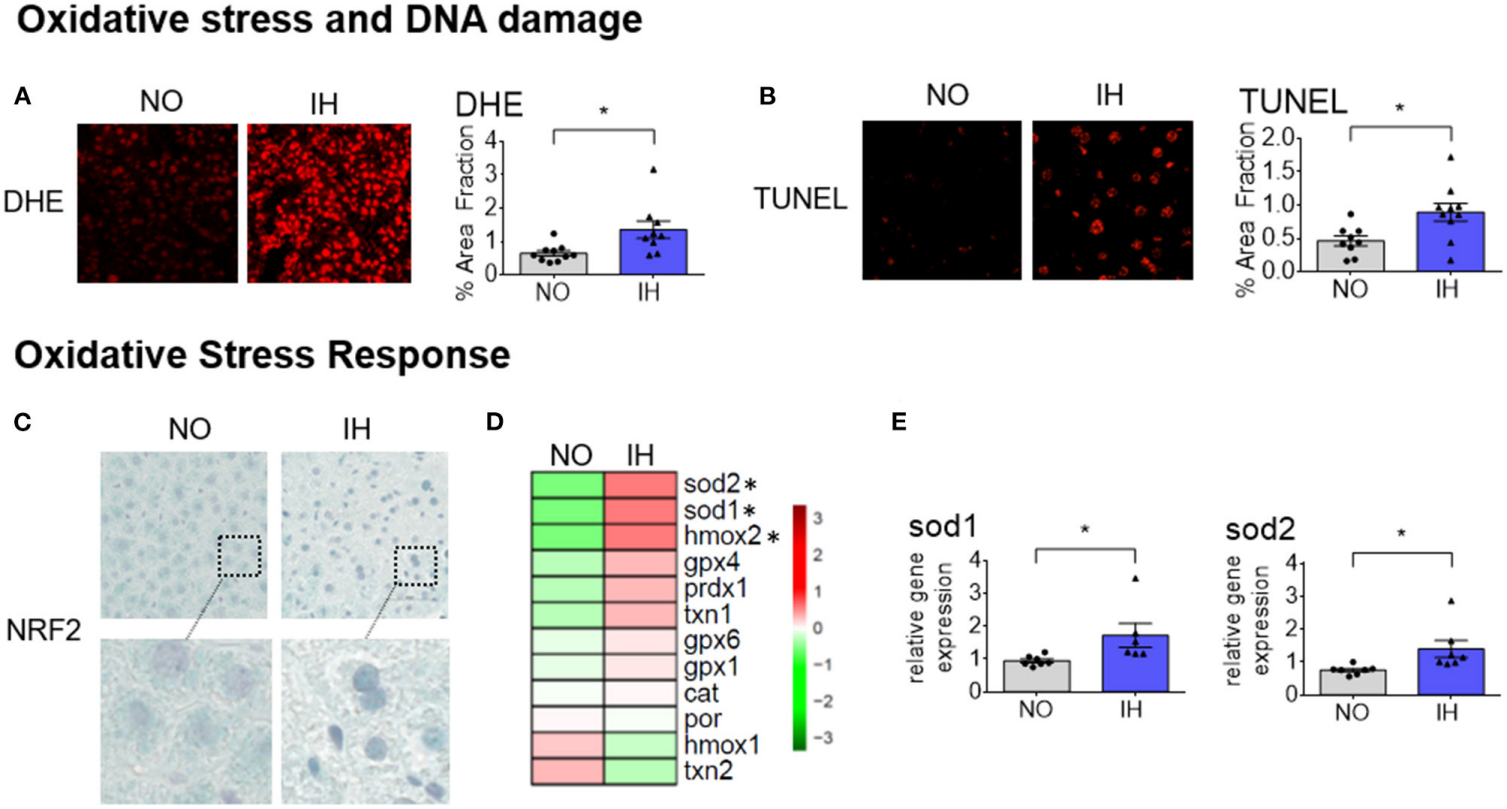
IH induces hepatic oxidative stress and DNA damage. **(A)** Levels of superoxide radicals and hydrogen peroxide assessed by dihydroethidium (DHE) staining. A comparative observation by confocal fluorescence microscopy of the ROS content in the liver sections from different groups. The fluorescent DHE signal was quantified and expressed as a percentage of total section area; two slides per animal (*n* = 5 biological replicates per group) were analyzed and are presented as mean + SEM. **(B)** Levels of DNA breaks determined by the TUNEL method. A comparative observation by confocal fluorescence microscopy of the DNA break content in the liver sections from different groups. The fluorescent TUNEL signal was quantified and expressed as a percentage of total section area; two slides per animal (*n* = 5 biological replicates per group) were analyzed and are presented as mean + SEM. **(C)** Representative Immunohistochemistry of NRF2 expression and localization in liver biopsies. Two slides per animal (*n* = 5 biological replicates per group) were analyzed. **(D)** Heatmap of representative gene expression profiles associated with oxidative stress response determined by quantitative real-time PCR. Gene expression was normalized to beta-actin and presented as mean + SEM (*n* = 7 biological replicates per group). Significance was calculated using Student's *t* test and *indicates *p*-value cut-off of 0.05. **(E)** Selected expression profiles of genes involved in antioxidant defense determined by quantitative real-time PCR. Gene expression was normalized to beta-actin and presented as mean + SEM (*n* = 7 biological replicates per group). Significance was calculated using Student's *t* test and *indicates *p*-value cut-off of 0.05.

Mitochondria are an important source of reactive oxygen species (ROS) produced by their ETC activity (22). Thus, modifications in mitochondrial respiration and NRF-dependent gene expression in the liver imply a cellular adjustment of the balance between oxidative stress and antioxidant defense. Therefore, we first examined cellular ROS production using dihydroethidium (DHE) staining, a specific marker for superoxide radicals, and observed a significant increase in the ROS level in the liver in the IH condition (Figure 5A). Since aberrant ROS production can cause oxidative modification of proteins and/or nucleic acids (such as DNA), we assessed the occurrence of DNA breaks using the terminal deoxynucleotide transferase [TdT]-mediated dUTP-digoxigenin nick-end labeling (TUNEL) method. Once again, IH caused a significant augmentation in DNA breaks in the liver (Figure 5B).

It is known that oxidative stress leads to NRF2 stabilization and translocation into the nucleus where it induces the expression of cytoprotective target genes. NRF2-dependant transcription had been highlighted in our transcriptomic dataset (Figure 1E) and accumulation of NRF2 in the nucleus was also observed by immunohistochemistry upon IH (Figure 5C). Accordingly, the expression of representative target genes implicated in antioxidant defenses was markedly increased upon IH (Figures 5D,E and Supplementary Figure 2). In particular, *Sod1* and *Sod2* genes encoding for the enzymes that catalyze the dismutation of superoxide radicals to molecular oxygen. Taken together, these results indicate that IH causes liver oxidative stress and induces a transcriptional adaptation involving the transcription factor NRF2.

## Discussion

Little is known about how IH impacts hepatic gene expression at a genome-wide level despite the fact that it has been clearly demonstrated that IH favors the progression of NAFLD (4, 6, 23). Here we employed unbiased transcriptomic analysis and revealed that IH induces a perturbation of nuclear respiratory factor (NRFs) dependent gene expression. While NRFs have overlapping function, NRF1 preferentially activates genes involved in mitochondrial respiratory function, whereas NRF2 activates genes required for the adaptive response to oxidants and electrophiles. These observations are of particular interest since mitochondrial dysfunction, oxidative stress and NRFs transcription factors have all been implicated in the pathogenesis of liver disease (24–27).

It is important to note that bulk liver transcriptomics likely contains response for other cell types and do not take in account cell type location in the liver. Therefore, it would have been compelling to complement this analysis with single cell and spatial transcriptomics to map precisely affected liver cells.

Data regarding NRF1 and IH are scarce (28). It had been suggested that NRF1 is up-regulated by IH and could be responsible for the increased mitophagy capacity (28). Our transcriptomic analysis demonstrated an upregulation of NRF1, potentially affecting mitochondrial dynamics and structure. Mitochondria are at the same time the main oxygen consumer and the primary ROS producer. Hence, it makes sense that rapid cycles of de-oxygenation and re-oxygenation mediated by IH can impact mitochondrial function. Only a few studies have explored the impact of sleep apnea and IH on mitochondrial metabolism in various tissues (29–33).

We showed that IH remodels mitochondrial structure and function. Specifically, we observed an increase in mitochondrial size possibly to maximize the surface of interaction with intracellular molecular oxygen. Surprisingly, we haven't found any differences either in the activity of isolated mitochondrial complex or in mitochondrial mass despite changes in mitochondrial respiration. It is important to note that the experimental protocol to determine individual complex activity do not explore complexes interactions occurring in intact mitochondria. Thus, the stability of mitochondrial mass might be explained by morphological modifications and remodeling of the interactions between mitochondrial complexes. The hepatic response to IH enlarging mitochondrial size instead of increasing mitochondrial number is, to our knowledge, a novel form of mitochondrial adaptation and warrants further investigations.

We revealed a marked increase in respiratory rate of isolated mitochondria under IH conditions, when fatty acids were used (Palmitoyl-carnitine). It is important to note that when lipids are used the respiratory exchange ratio is lowered and this change in mitochondrial metabolism could be an adaptative strategy under IH conditions. This adjustment is also associated with an increase in the rate-limiting protein involved in mitochondrial fatty acid oxidation suggesting an overall remodeling of mitochondrial fuel usage. Interestingly, sleep apnea and IH are associated with higher than normal levels of circulating fatty acids possibly originating from adipose tissue lipolysis and excessive ectopic fat in SAS (34–36). Therefore, it is plausible that the liver adapts its metabolism to increased flux of fatty acids, in order to maintain the organism's homeostasis. We could not exclude that IH also rewire the usage of other fuels such as glucose and amino acids. It would be engaging to use radioactive probes, along with pharmaceutical and surgical procedures to test these complementary hypotheses *in vivo*.

SAS is characterized by oxidative stress triggered by intermittent hypoxia (37, 38). In NAFLD, oxidative stress is a well-recognized contributor to the progression of liver injury. Considering that SAS is highly prevalent in NAFLD patients, it is conceivable that IH is probably an underestimated producer of ROS in the NAFLD population (39). Previous literature (15, 40) is suggesting that fatty acid usage for mitochondrial respiration favors the production of ROS by the reverse electron flux from the mitochondrial complex 1.

Recently, it has been demonstrated in NASH patients that NRF2 activation is correlated with liver inflammation (41). In response to oxidative stress, transcriptional activity of NRF2 be increase in order to generate an antioxidant response (42).

After 2 weeks of IH and before any histological sign of liver injury (data not shown), our transcriptomic analysis revealed an amplified contribution of NRF2. Moreover, our immunohistochemistry experiments showed the accumulation of NRF2 in the nucleus upon IH. We also found a higher expression of some representative target genes implicated in antioxidant defenses. This is consistent with NRF2 activation having been shown to be protective against the progression of liver disease by ameliorating fibrogenesis in mice (41). Therefore, it is possible that NRF2 pathway is activated to counteract deleterious role of IH and maintain liver homeostasis in the early stage of NAFLD. The role of NRF2 later in the progression of NAFLD under IH remains to be investigated. It would have been of interest to assess hepatic NRF2 signaling in the course of NAFLD progression in apneic patients.

Although NRFs are master regulators of mitochondrial metabolism and antioxidant defenses, we could not exclude the implication of others factors such as ATF4, HIF1A and PGC1A. For example, the stress response transcription factor ATF4 is pointed out by our TFBS analysis (Figure 1F). ATF4 is well known to be induced by numerous stress such as hypoxia, ER stress, oxidative stress and we have previously shown that ATF4 is contributing to IH dependent cell death in the heart (12). Whether these factors work together to regulate hepatic adaptation/maladaptation to IH needs to be further investigated.

Our findings contribute to the understanding of the mechanisms underlying NAFLD development and progression during SAS and provide a rationale for novel therapeutic targets and biomarker discovery.

## Data Availability Statement

The datasets presented in this study can be found in online repositories. The names of the repository/repositories and accession number(s) can be found below: https://www.ncbi.nlm.nih.gov/geo/, GSE104128.

## Ethics Statement

The animal study was reviewed and approved by the Institutional Ethics Committee for Animal Care and Use (Cometh 12) and the French Ministry of Research (APAFIS# 15156-2018051615245109).

## Author Contributions

JG and J-LP conceived and designed the study and drafted the manuscript. VP, EM, and JG performed bioinformatic analysis and produced the graphics. JG, MG, and EL performed RNA extractions and analysis. SB performed the immunohistochemical analysis. AB and KP-G performed the electron microscopy. FL and GV performed mitochondrial isolations and characterizations. A-LB, CA, EB, KC, DG, and JA-W provided technical support for project design and data analysis and participated in interpretation. All authors critically revised the manuscript for important intellectual content and approved the version for publication.

## Funding

The HP2 laboratory was supported by the Institut National de la Santé et de la Recherche Médicale (INSERM), the University of Grenoble-Alpes (UGA), the Fondation Agir Pour les Maladies Chroniques (APMC, Project Temporize Liver), the Agence Nationale pour la Recherche (ANR, Project Temporize, ANR-19-CE14-0037-01), and the ANR Initiatives d'Excellence de l'UGA (ANR-IDEX-UGA, Project LiFE, 15-IDEX-0002). This work has been partially supported by UGA e-health chair and MIAI @ university Grenoble Alpes (ANR-19-P3IA-0003). JG was the recipient of the postdoctoral fellowship from the ANR-IDEX-UGA Initiative de Recherche Stratégique (ANR-IDEX-UGA-IRS, Project HypoClock). GV received a grant from Société Francophone du Diabète. EM work was supported by a postdoctoral fellowship from Fondation ARC. MG was financed by the Medical Research grant from Montpellier-Nimes Medical School. KC has received support from EU litmus grant and French foundation for medical research. JA-W received a grant from Bettencourt Shueller foundation.

## Conflict of Interest

The authors declare that the research was conducted in the absence of any commercial or financial relationships that could be construed as a potential conflict of interest.

## Publisher's Note

All claims expressed in this article are solely those of the authors and do not necessarily represent those of their affiliated organizations, or those of the publisher, the editors and the reviewers. Any product that may be evaluated in this article, or claim that may be made by its manufacturer, is not guaranteed or endorsed by the publisher.

## Supplementary Material

Supplementary Primers List Primers used for RT-qPCR.

Supplementary Figure 1 Number of mice used in the study.

Supplementary Figure 2 IH rewires hepatic gene expression related to mitochondrial function. Heatmap of mitochondrial function related genes found in the Gene Ontology (GO) analysis of the hepatic transcriptome (related to Figure 1D) showing individual scores per sample per group extracted from the microarray analysis. The following GO are presented: (top) GO Cellular Component Mitochondrion GO0005739 and (bottom) KEGG Oxidative Phosphorylation KO00190.

Supplementary Figure 3 IH rewires hepatic gene expression related to mitochondrial dynamics. Heatmap of the Gene Ontology (GO) analysis of the hepatic transcriptome showing individual scores per sample per group extracted from the microarray analysis. The following GO are presented: (top) GO Biological Pathway Mitophagy GO0000423 and (middle) GO Biological Pathway Fusion GO0008053 and (bottom) GO Biological Pathway Fission GO0000266.

Supplementary Figure 4 Mitochondrial number evaluation. Mitochondrial number was evaluated by RT-qPCR by determining the ratio of mitochondrial DNA over nuclear DNA (*n* = 4 biological replicates per group) and presented as mean + SEM.

Supplementary Figure 5 Gene expression profiles determined by quantitative real-time PCR. Gene expression was normalized to beta-actin and presented as mean + standard error of mean (SEM, *n* = 7 biological replicates per group). Significance was calculated using Student's t test and *indicates *p*-value cut-offs of 0.05.
