# Supplementary figures and images for: Intermittent Hypoxia Rewires the Liver Transcriptome and Fires up Fatty Acids Usage for Mitochondrial Respiration
Source: Front Med (Lausanne). 2022 Feb 18;9:829979. doi: 10.3389/fmed.2022.829979 (PMC8894659; doi:10.3389/fmed.2022.829979)

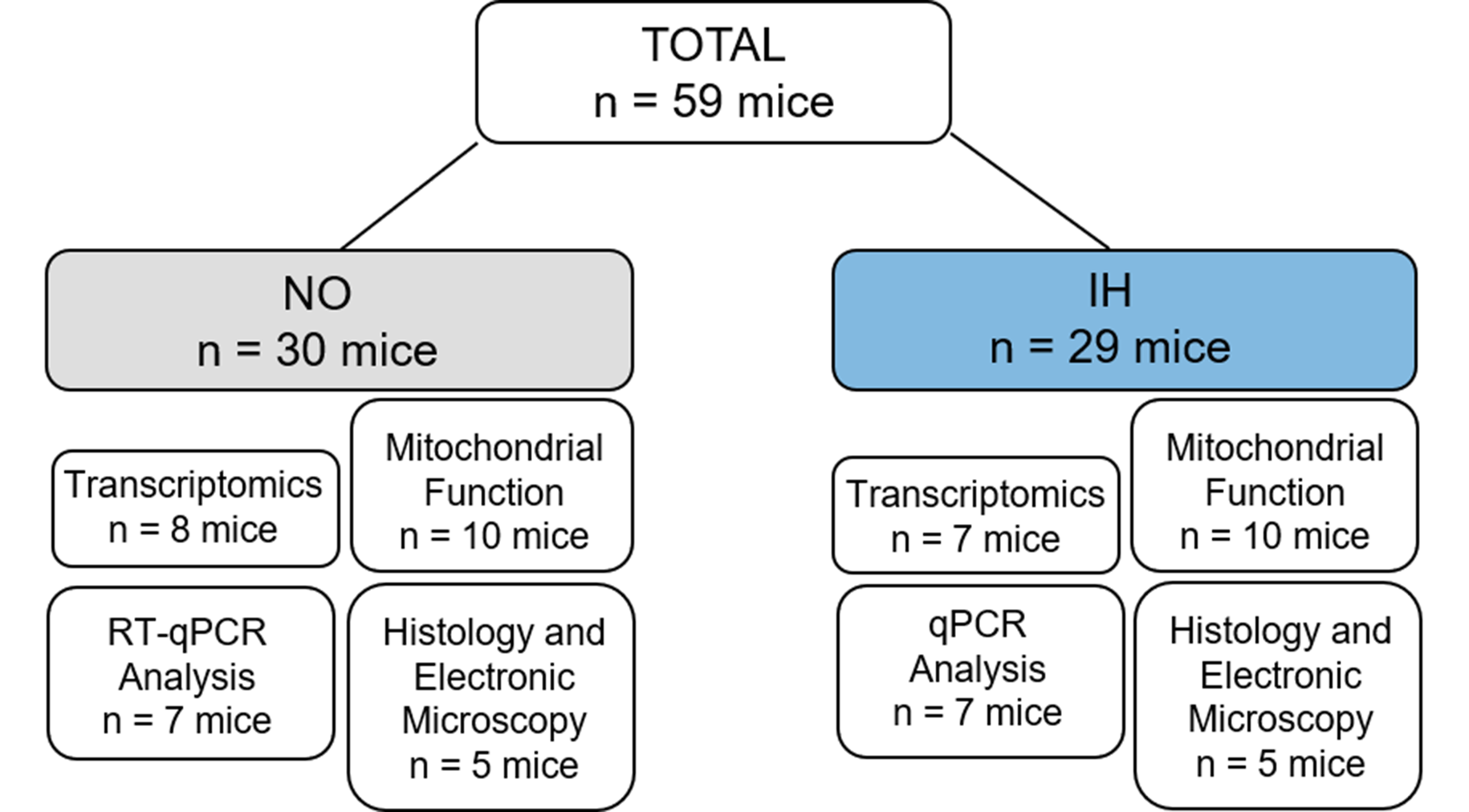

Supplement: Supplementary Figure 1 — Number of mice used in the study. [file Image_1.TIF]

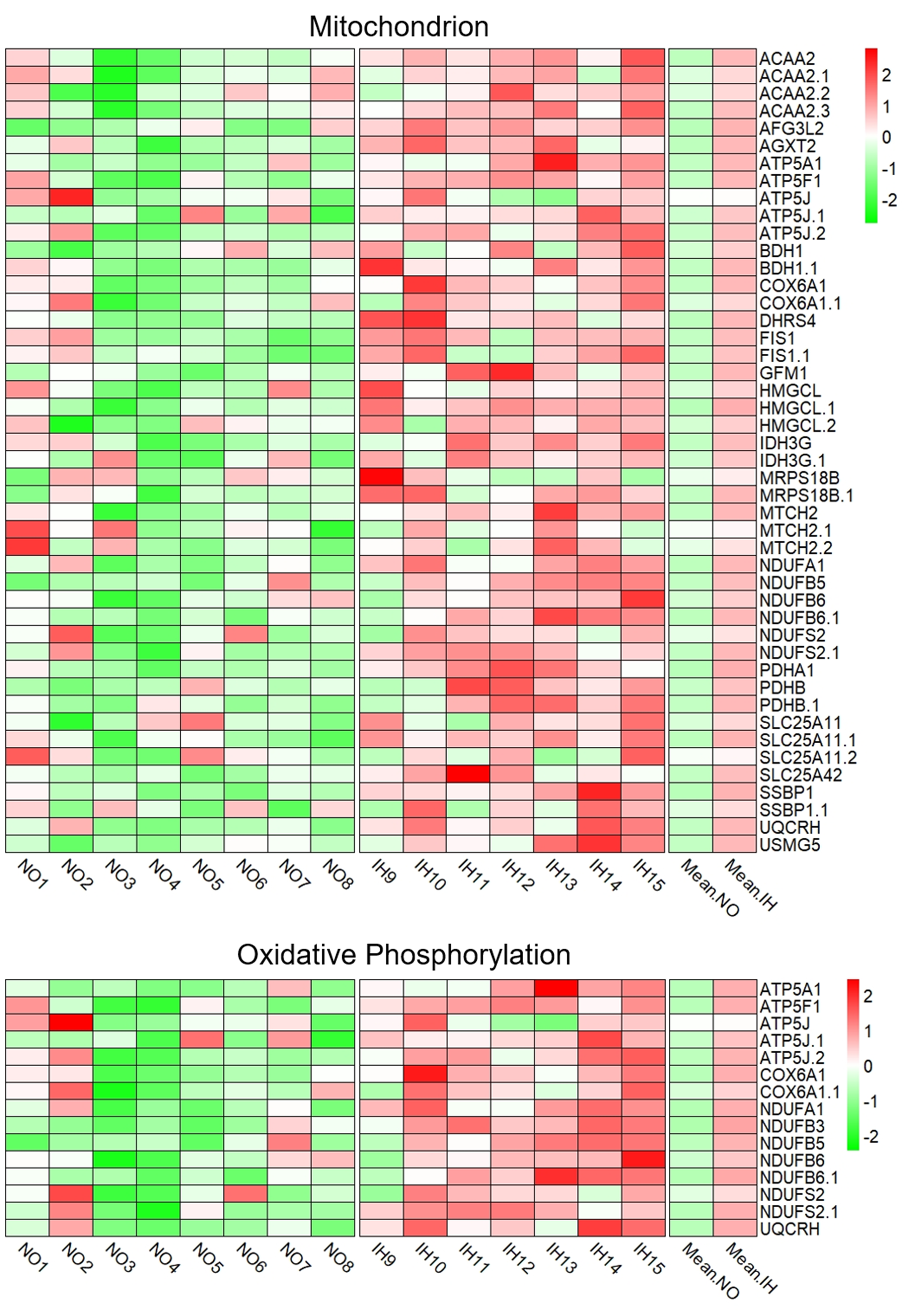

Supplement: Supplementary Figure 2 — IH rewires hepatic gene expression related to mitochondrial function. Heatmap of mitochondrial function related genes found in the Gene Ontology (GO) analysis of the hepatic transcriptome (related to Figure 1D) showing individual scores per sample per group extracted from the microarray analysis. The following GO are presented: (top) GO Cellular Component Mitochondrion GO0005739 and (bottom) KEGG Oxidative Phosphorylation KO00190. [file Image_2.TIF]

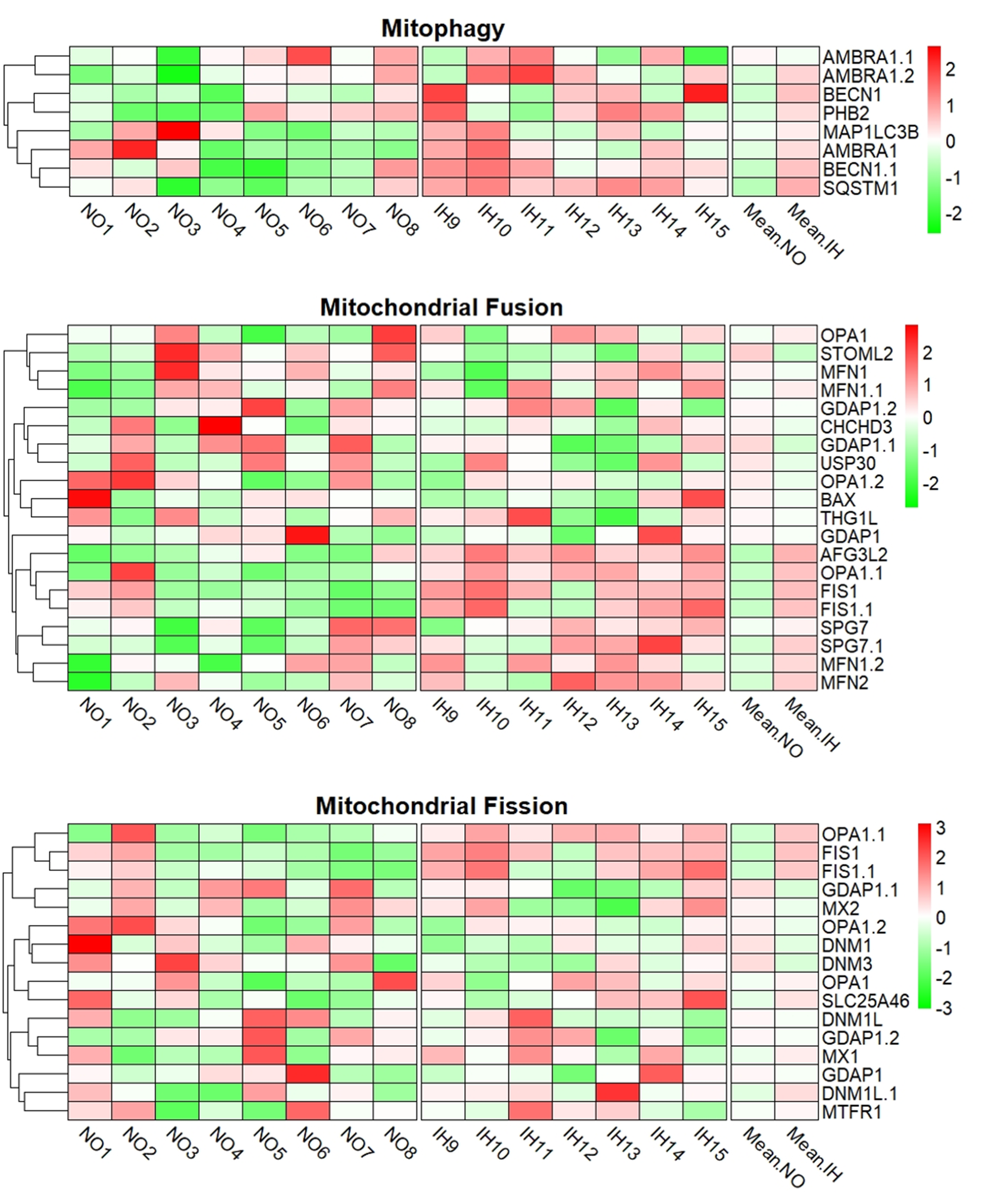

Supplement: Supplementary Figure 3 — IH rewires hepatic gene expression related to mitochondrial dynamics. Heatmap of the Gene Ontology (GO) analysis of the hepatic transcriptome showing individual scores per sample per group extracted from the microarray analysis. The following GO are presented: (top) GO Biological Pathway Mitophagy GO0000423 and (middle) GO Biological Pathway Fusion GO0008053 and (bottom) GO Biological Pathway Fission GO0000266. [file Image_3.TIF]

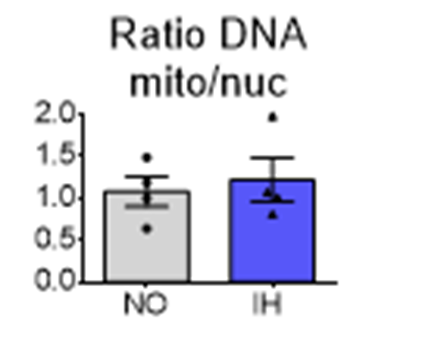

Supplement: Supplementary Figure 4 — Mitochondrial number evaluation. Mitochondrial number was evaluated by RT-qPCR by determining the ratio of mitochondrial DNA over nuclear DNA (n = 4 biological replicates per group) and presented as mean + SEM. [file Image_4.TIF]

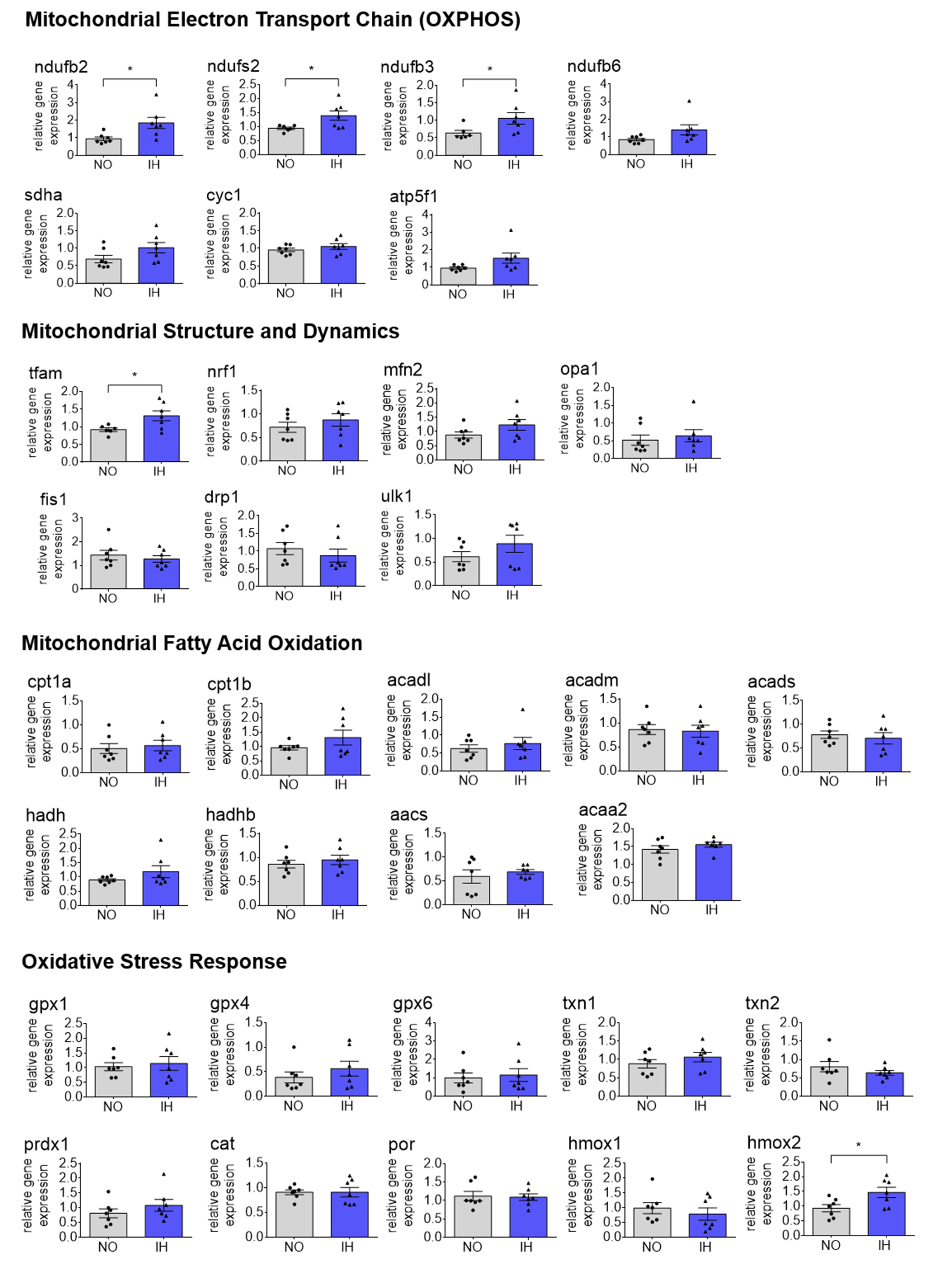

Supplement: Supplementary Figure 5 — Gene expression profiles determined by quantitative real-time PCR. Gene expression was normalized to beta-actin and presented as mean + standard error of mean (SEM, n = 7 biological replicates per group). Significance was calculated using Student's t test and *indicates p-value cut-offs of 0.05. [file Image_5.TIF]
